# High triglyceride levels increase the risk of diabetic microvascular complications: a cross-sectional study

**DOI:** 10.1186/s12944-023-01873-5

**Published:** 2023-07-31

**Authors:** Jiahang Li, Lei Shi, Guohong Zhao, Fei Sun, Zhenxing Nie, Zhongli Ge, Bin Gao, Yan Yang

**Affiliations:** 1 Department of Pharmacy, Tangdu Hospital of Air Force Medical University, Xi’an, 710038 China; 2 Department of Endocrinology, Tangdu Hospital of Air Force Medical University, Xi’an, 710038 China; 3 Department of Pharmacy, Hanzhong Municipal People’s Hospital, Hanzhong, 723000 China

**Keywords:** Triglyceride, Diabetes mellitus, Diabetic nephropathy, Diabetic neuropathy, Diabetic retinopathy

## Abstract

**Background:**

The prevalence of microvascular complications in type 2 diabetes mellitus (T2DM) is increasing. The effect of lipid profiles on diabetic microvascular complications remains debated. This research aimed to study the correlation between lipid profiles and microvascular complications.

**Methods:**

This retrospective cross-sectional study included 1096 T2DM patients. The patients were divided into the control, diabetic retinopathy (DR), nephropathy (DKD), and peripheral neuropathy (DPN) groups based on the existence of corresponding complications. The lipid profiles were analyzed, and the effect on complications was assessed by logistic regression.

**Results:**

Compared with the control group, the diabetic microvascular complications group had a higher dyslipidemia rate. The rate of high TGs increased significantly with an increasing number of complications. High TG levels contributed to the risk of DKD, DR, and DPN [odds ratios (ORs): 2.447, 2.267, 2.252; 95% confidence interval: 1.648–3.633, 1.406–3.655, 1.472–3.445]. In the age (years) > 55, T2DM duration (years) > 10, and HbA1c (%) ≥ 7 groups, the risk of high TGs was higher for DKD (ORs: 2.193, 2.419, 2.082), DR (ORs: 2.069, 2.317, 1.993), and DPN (ORs: 1.811, 1.405, 1.427).

**Conclusion:**

High TG levels increase the risk of diabetic microvascular complications, and patients with older age, longer T2DM duration, and higher HbA1c levels are recommended to keep lipid levels more strictly.

## Introduction

Over the last 50 years, type 2 diabetes mellitus (T2DM) has been increasing in number, and according to current models it is expected that nearly 700 million people worldwide will be affected by 2045 [1]. Along with cardiovascular disease and tumors, T2DM is now another chronic noncommunicable disease that poses a serious health risk to the public [2, 3]. Microvascular complications represent the most prevalent chronic complication of T2DM and can cause damage and dysfunction of the microvasculature. Diabetic retinopathy (DR), diabetic peripheral neuropathy (DPN), and diabetic nephropathy (DKD) contribute to microvascular complications, thereby affecting the prognosis of the disease. Microvascular complications are the essential factors of death and disability in T2DM, seriously affect the patients’ physical and mental health, and bring a great medical cost to society [4].

Hyperglycemia and hypertension are important causative drivers of microvascular complications [5, 6]. However, some patients with early-stage diabetes still progress to diabetic microvascular complications even when blood glucose and blood pressure are under control [4, 7, 8]. To address this challenge, new prevention strategies need to be developed. Lipid metabolism is associated with diabetic microvascular complications. Although there have been many studies correlating diabetic microvascular complications with lipid profiles, the findings have been highly debated. In addition, previous research from our group found that the dyslipidemia rate is very high in patients with diabetic complications [9]. However, it is unclear which lipid profiles have a greater impact on diabetic complications.

Hence, the aim of this study was to explore exploring the relationship between lipid profiles and DKD, DR, and DPN.

## Methods

### Data collection

**Fig. 1:**
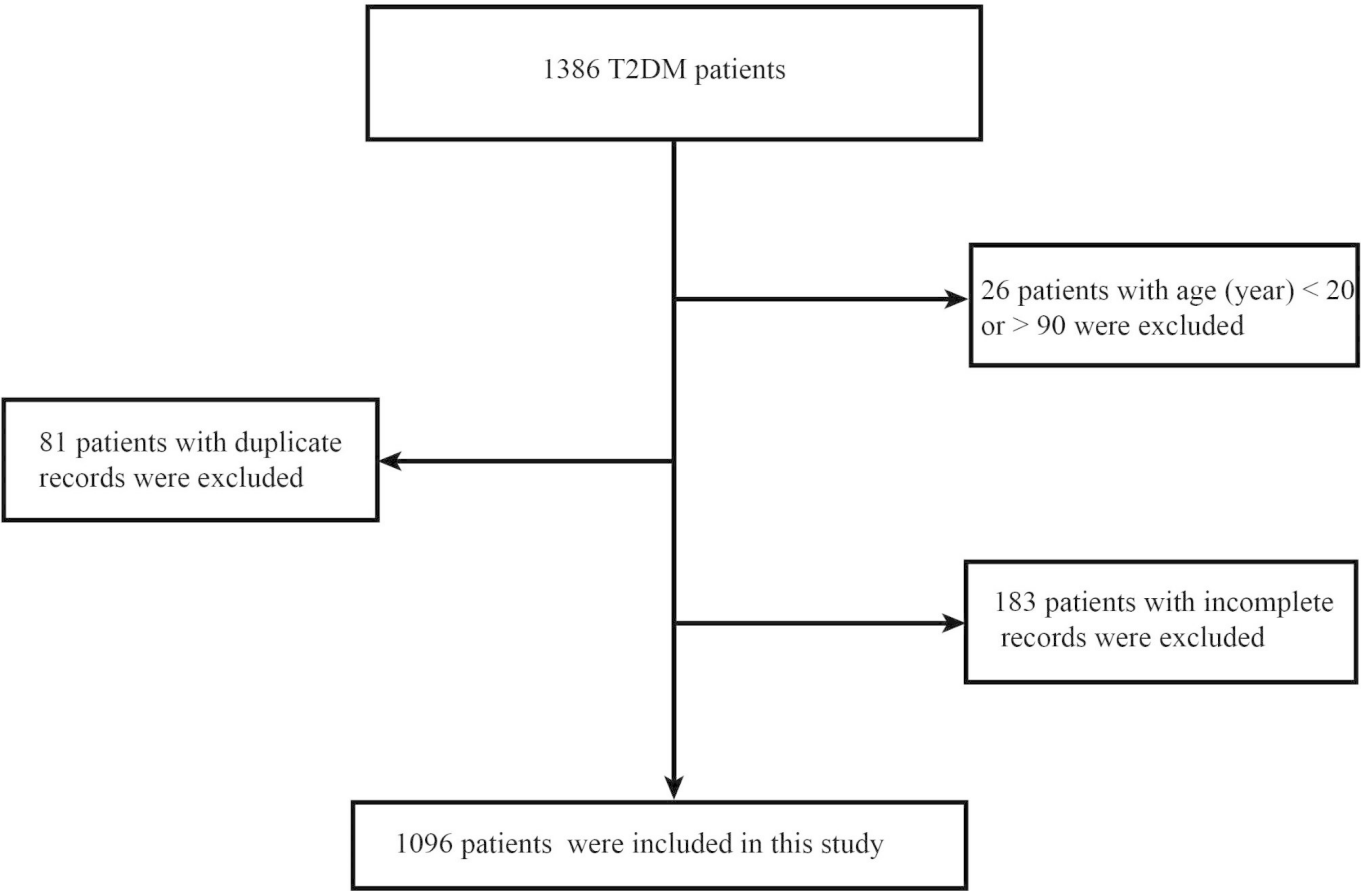
Flowchart for exclusion criteria

The investigators collected medical records of T2DM inpatients from March 2021 to February 2022 in Tangdu Hospital. A total of 1386 participants from Northwest China were selected for inclusion in the cross-sectional retrospective study. Inclusion criteria included the following: (1) clear type 2 diabetes (not type 1, gestational diabetes or specific type of diabetes); (2) age between 20 and 90 years; and (3) a serum lipid test with complete data during hospitalization. Figure 1 shows the inclusion criteria in detail. The clinical information encompassed sex, age, T2DM duration, body mass index (BMI), lipid-lowering medications, hypertension, DKD, DR, and DPN. Anthropometric measurements included serum lipid profiles and glycated hemoglobin (HbA1c) levels. The research was approved by the Tangdu Hospital Ethics Committee (reference number: K202205-07).

### Definitions and groups

Clinical diagnosis of dyslipidemia and T2DM was based on the Chinese guidelines [10] and the World Health Organization’s [1], respectively. The cutoff values for high total cholesterol (TC), high triglyceride (TG), high low-density lipoprotein cholesterol (LDL-C), and low high-density lipoprotein cholesterol (HDL-C) were 6.22, 2.26, 4.14, and 1.04 mmol/L, respectively. Any patient with one of the above indicators or currently taking lipid-lowering medications was diagnosed with dyslipidemia [11]. Diagnosis of DKD was based on glomerular filtration rate (GFR) less than 60 mL/min/1.73 m^2^ or urinary albumin/creatinine ratio (ACR) more than 30 mg/g > 3 months with the exclusion of other chronic kidney diseases [1]. DR was defined by an ophthalmologist based on dilated fundoscopy in accordance with the International Clinical Classification Criteria [1, 7]. The diagnosis of DPN was based on a clear history of diabetes, a clear temporal relationship with diabetes, and a consistent clinical presentation with DPN (pain, numbness, and paresthesia) [1, 12]. The control group was diabetic patients without microvascular complications. The DKD, DR, and DPN groups were classified depending on the existence of the corresponding complications. The patients were also divided into groups 0, 1, 2, and 3 according to their number of microvascular complications.

### Statistical analysis

SPSS Statistics 26 was used to analyze all data. *P* < 0.05 (two-sided) was treated as a significant difference.The variables were expressed as the mean ± standard deviation or numbers (percentages). In addition, the comparisons between groups were conducted with independent sample *t* test (continuous variables) or Chi-square test (categorical variables).

The impact of dyslipidemia or lipid profiles on microvascular complications was evaluated by applying multiple logistic regression analysis. In addition, the effects of high TG levels on microvascular complications were investigated by subgroup analysis, including: gender, age, T2DM duration, HbA1c (%) level and BMI. TG levels were grouped in quintiles according to TG concentration: quartile 1 (Q1): 0–1.12 mmol/L, quartile 2 (Q2): 1.13–1.59 mmol/L, quartile 3 (Q3): 1.60–2.06 mmol/L, quartile 4 (Q4): 2.07–2.98 mmol/L, quartile 5 (Q5): 2.99–14.93 mmol/L. The multiple logistic regression was used to analyze the effect of different TG levels on microvascular complications.

## Results

### General characteristics

Table 1 shows the patients’ characteristics. The patients were 55.23 ± 13.39 years old, with 597 patients being over 55 years old; and 745 being male. The T2DM duration was 9.48 ± 7.19 years, and 292 patients had an HbA1c (%) of less than 7. The average BMI was 26.00 ± 3.73 kg/m^2^. DKD, DR, and DPN were present in 38.9%, 25.0% and 32.8% of this population, respectively. Hypertension and lipid-lowering medication use accounted for 46.0% and 56.3% of the population, respectively. Meanwhile, the average levels of TC, TG, HDL-C and LDL-C were 4.42 ± 1.10, 2.22 ± 1.52, 1.07 ± 0.37, and 2.51 ± 0.94 mmol/L, respectively.

**Table 1:** General characteristics

	Patients (N = 1096)
Female	351 (32.0%)
Age (years)	55.23 ± 13.39
> 55	597 (54.0%)
HbA1c (%)	8.60 ± 2.20
< 7	292 (26.6%)
T2DM duration (years)	9.48 ± 7.19
> 10	429 (39.1%)
BMI (kg/m^2^)	26.00 ± 3.73
< 24	777 (70.9%)
DKD	426 (38.9%)
DR	274 (25.0%)
DPN	359 (32.8%)
Hypertension	504 (46.0%)
Lipid-lowering drug	617 (56.3%)
Lipid profile (mmol/L)	
TC	4.42 ± 1.10
TG	2.22 ± 1.52
HDL-C	1.07 ± 0.37
LDL-C	2.51 ± 0.94

Abbreviations: HbA1c, glycated hemoglobin; T2DM, type 2 diabetes mellitus; BMI, body mass index; DKD, diabetic kidney disease; DR, diabetic retinopathy; DPN, diabetic peripheral neuropathy; TC, total cholesterol; TG, triglyceride; HDL-C, high-density lipoprotein cholesterol; LDL-C, low-density lipoprotein cholesterol.

### Comparisons of patient characteristics with and without microvascular complications

There were some variations in the clinical indicators between the control and DKD, DR, or DPN groups (Table 2). Age, hypertension rate, and T2DM duration were significantly higher in the DKD, DR, and DPN groups than in the control group. Meanwhile, in the DKD, DR or DPN group, dyslipidemia prevalence was also significantly higher (control, DKD, DR, DPN group: 62.1%, 88.7%, 89.8%, and 90.3%). Compared to the control group, TG and TC levels were higher (TG, 2.10 ± 1.36 vs. 2.50 ± 1.76 mmol/L; TC, 4.34 ± 0.92 vs. 4.65 ± 1.25 mmol/L) and HDL-C levels were significantly decreased (1.11 ± 0.30 vs. 1.04 ± 0.27 mmol/L) in the DKD group.

**Table 2:** Comparisons of characteristics among the different groups

	Control	DKD	DR	DPN
N (%)	364 (33.2%)	426 (38.9%)	274 (25.0%)	359 (32.8%)
Male	242 (66.5%)	317 (74.4%)^*^	181 (64.1%)	233 (64.9%)
Age (years)	48.50 ± 13.50	57.17 ± 12.55^***^	60.43 ± 10.86^***^	61.18 ± 10.47^***^
> 55	124 (34.1%)	252 (59.2%)^***^	194 (70.8%)^***^	267 (74.4%)^***^
HbA1c (%)	8.89 ± 2.43	8.54 ± 2.09	8.41 ± 2.13	8.29 ± 2.08
< 7	93 (25.5%)	110 (25.8%)	76 (27.7%)	106 (29.5%)
T2DM duration (years)	5.43 ± 5.02	11.28 ± 7.32^***^	13.63 ± 7.21^***^	12.84 ± 6.72^***^
> 10	55 (15.1%)	214 (50.2%)^***^	177 (64.6%)^***^	217 (60.4%)^***^
BMI (kg/m^2^)	26.02 ± 4.05	26.39 ± 3.65	25.55 ± 3.35	25.69 ± 3.55
< 24	262 (72.0%)	313 (73.5%)	182 (66.4%)	237 (66.0%)
Hypertension	93 (25.5%)	273 (64.1%)^***^	176 (64.2%)^***^	186 (51.8%)^***^
Lipid profile (mmol/L)				
TC	4.34 ± 0.92	4.65 ± 1.25^***^	4.49 ± 1.29	4.31 ± 1.12
TG	2.10 ± 1.36	2.50 ± 1.76^***^	2.21 ± 1.35	2.12 ± 1.33
HDL-C	1.11 ± 0.30	1.04 ± 0.27^**^	1.06 ± 0.54	1.05 ± 0.50
LDL-C	2.61 ± 0.87	2.57 ± 1.02	2.42 ± 1.03	2.36 ± 0.92
Dyslipidemia	226 (62.1%)	378 (88.7%)^***^	246 (89.8%)^***^	324 (90.3%)^***^
High TC	18 (4.9%)	46 (10.8%)^*^	25 (9.1%)^*^	18 (5.0%)
High TG	94 (25.8%)	197 (46.2%)^***^	122 (44.5%)^***^	144 (40.1%)^***^
Low HDL-C	152 (41.8%)	240 (56.3%)^***^	160 (58.4%)^***^	212 (59.1%)^***^
High LDL-C	11 (3.0%)	24 (5.6%)	14 (5.1%)	14 (3.9%)

*P* values were for chi-square tests (categorical variables) or independent-samples *t* tests (continuous variables) among the groups (vs. control, ^*^P < 0.05, ^**^P < 0.01, ^***^P < 0.001).

### Differences in the patients’ characteristics depending on the number of complications

As shown in Table 3, the patients’ characteristics were different in different groups. With the increase in the number of complications, the rates of hypertension, age > 55 years, T2DM duration > 10 years, and dyslipidemia gradually increased. The increasing trend in high TGs was most pronounced as the number of complications increased; the rates of high TGs were 25.8%, 35.6%, 45.7%, and 59.4% in the 0, 1, 2, and 3 complication groups, respectively.

**Table 3:** Patients’ characteristics depending on the complication numbers

	the complication number			
	0	1	2	3
N (%)	364 (33.2%)	469 (42.8%)	199 (18.2%)	64 (5.8%)
Male	242 (66.5%)	319 (68.0%)	140 (70.4%)	44 (68.8%)
Age (years) > 55^***^	124 (34.1%)	284 (60.6%)	138 (69.3%)	267(79.7%)
HbA1c (%) < 7	93 (25.5%)	123 (26.2%)	59 (29.6%)	17 (26.6%)
T2DM duration (years) > 10^***^	55 (15.1%)	191 (40.7%)	132 (66.3%)	51 (79.7%)
BMI (kg/m^2^) < 24	262 (72.0%)	339 (72.3%)	135 (67.8%)	41 (64.1%)
Hypertension ^***^	93 (25.5%)	239 (51.0%)	120 (60.3%)	52 (81.3%)
Dyslipidemia^***^	226 (62.1%)	414 (88.3%)	177 (88.9%)	60 (93.8%)
High TC^*^	18 (4.9%)	34 (7.2%)	11 (5.5%)	11 (17.2%)
High TG^***^	94 (25.8%)	167 (35.6%)	91 (45.7%)	38 (59.4%)
Low HDL-C^***^	152 (41.8%)	269 (57.4%)	116 (58.3%)	37 (57.8%)
High LDL-C^*^	11 (3.0%)	17 (3.6%)	7 (3.5%)	7 (10.9%)

*P* values were for chi-square tests (categorical variables) among groups (^*^P < 0.05, ^**^P < 0.01, ^***^P < 0.001)

### Multivariate risk assessment for microvascular complications

Multivariate logistic regression was employed to evaluate the effects of lipid profiles on microvascular complications (Table 4). High TG levels and DKD, DR, and DPN were significantly associated after adjusting for HbA1c level, hypertension, age, sex, T2DM duration, BMI, and medical treatment. The odds ratios (ORs) of high TG levels were 2.447, 2.267 and 2.252 [95% confidence interval (CI)1.648–3.633, 1.406–3.655, 1.472–3.445] for the increased risk of DKD, DR, and DPN, respectively.

**Table 4:** Multivariate risk assessment for microvascular complications

Variables	DKD		DR		DPN	
	OR (95% CI)	*P* value	OR (95% CI)	*P* value	OR (95% CI)	*P* value
High TC	1.640 (0.712–3.779)	0.245	1.353 (0.532–3.441)	0.526	0.489 (0.177–1.349)	0.167
High TG	2.447 (1.648–3.633)	< 0.001	2.267 (1.406–3.655)	0.001	2.252 (1.472–3.445)	< 0.001
Low HDL-C	1.358 (0.930–1.982)	0.113	1.548 (0.979–2.448)	0.061	1.409 (0.946–2.098)	0.091
High LDL-C	1.293 (0.436–3.829)	0.643	1.468 (0.430–5.014)	0.540	2.060 (0.667–6.361)	0.209

Adjusted for age, sex, duration of T2DM, medical treatment, hypertension, BMI, and HbA1c level.

### The effects of microvascular complications from high TG by subgroup analysis

**Fig. 2:**
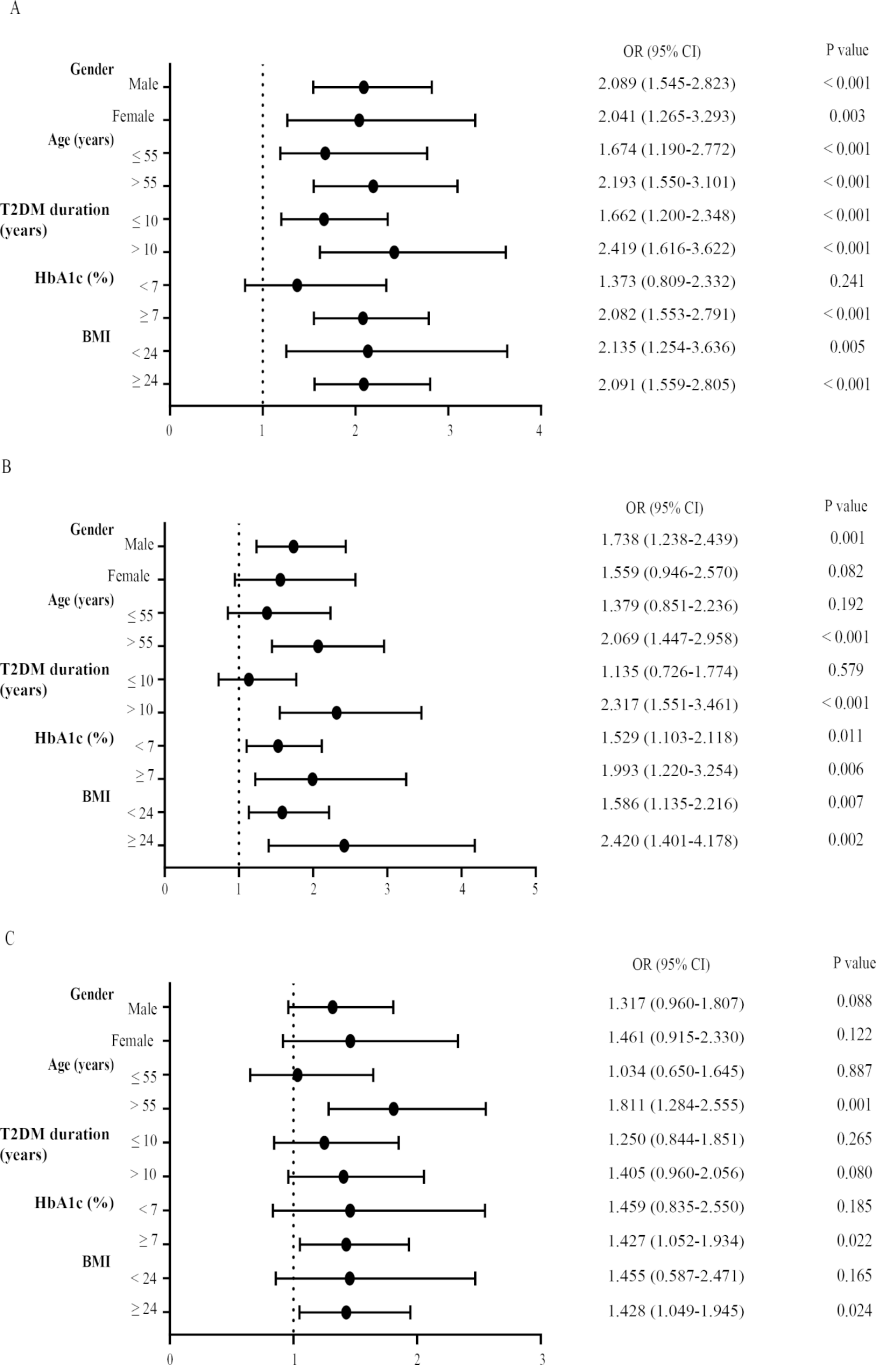
The ORs of high TG levels by gender, age, T2DM duration, HbA1c and BMI for the DKD (**A**), DR (**B**), and DPN (**C**)

The effects of high TG levels on microvascular complications are different between different subgroups (Fig. 2). The risk of high TG levels did not show the significant difference by different gender and BMI. However, in age (years) > 55 group, the ORs were 2.193 (1.550–3.101), 2.069 (1.447–2.958), and 1.811 (1.284–2.555) for the DKD, DR and DPN, respectively. The ORs in T2DM duration (years) > 10 group were 2.419 (1.616–3.622), 2.317 (1.551–3.461), and 1.405 (0.960–2.056) for the DKD, DR and DPN, respectively. The ORs in HbA1c (%) ≥ 7 group were 2.082 (1.553–2.791), 1.993 (1.220–3.254), and 1.427 (1.052–1.934) for the DKD, DR and DPN, respectively. The age (years) > 55, T2DM duration (years) > 10, and HbA1c (%) ≥ 7 all significantly increased the risk of high TG levels for the microvascular complications.

### Risk assessment of different TG levels for microvascular complications

**Fig. 3:**
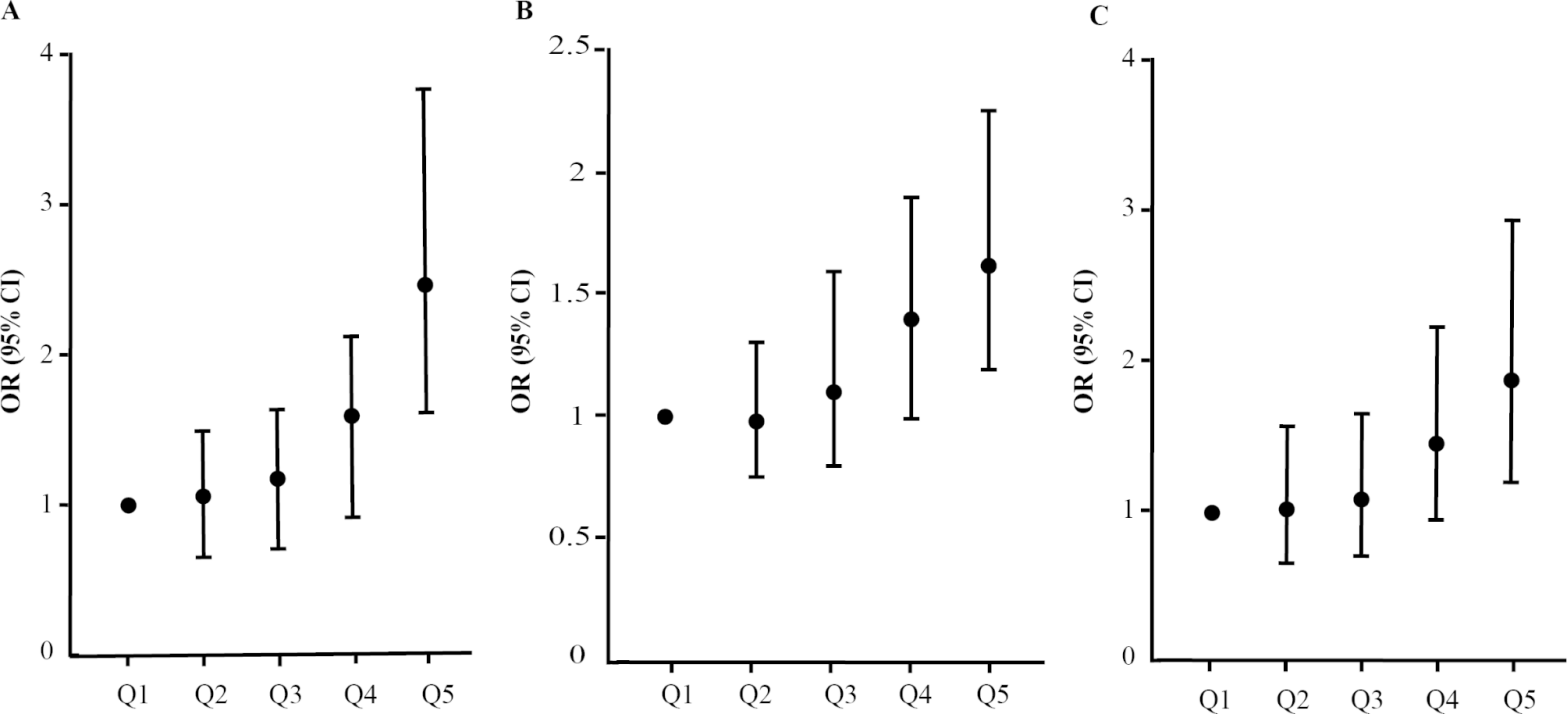
The risk assessment of different TG levels (Q1: 0–1.12 mmol/L, Q2: 1.13–1.59 mmol/L, Q3: 1.60–2.06 mmol/L, Q4: 2.07–2.98 mmol/L, Q5: 2.99–14.93 mmol/L) for the DKD (**A**), DR (**B**), and DPN (**C**). Adjusted for age, gender, duration of T2DM, medical treatment, hypertension, BMI, and HbA1c level

The effects of different TG levels on microvascular complications are different (Fig. 3). From the Q2 to Q3 groups, the ORs of high TGs were 1.085 to 1.170, 0.981 to 1.104, and 1.028 to 1.096 for DKD, DR, and DPN, respectively. However, in the Q4 and Q5 groups, the ORs were significantly higher. From the Q4 to Q5 groups, the ORs were 1.586 to 2.457 in the DKD group, 1.407 to 1.631 in the DR group, and 1.473 to 1.899 in the DPN group. The results showed that high TG levels significantly increased the risk of microvascular complications.

## Discussion

Diabetic dyslipidemia and microvascular complications have a close relationship. In this cross-sectional survey, high TG levels showed a significant correlation with the risk of diabetic microvascular complications, and the effects were more significant in patients with age (years) > 55, T2DM duration (years) > 10, and HbA1c (%) ≥ 7.

Microvascular complications represent the most prevalent chronic complication of T2DM and can cause damage and dysfunction of the microvasculature. DR, DPN, and DKD are the most common microvascular complications in diabetes [13, 14]. According to a survey, the prevalence of DR, DPN, and DKD is 25–40%, 19.7–51.7% and 25–40%, respectively [15–17]. In this study, the prevalence of DKD, DR, and DPN in T2DM patients was 38.9%, 25.0%, and 32.8%, respectively. The result was consistent with other studies and indicated that the situation of diabetic complications in China was very serious.

There are close relationships between dyslipidemia and microvascular complications. Giuseppina T, Frank M et al. indicated that high TG was the main feature for the development of DKD, and the rate of high TG was higher in DKD and DR patients [5, 18, 19]. The TG/HDL ratio was higher in patients with complicated T2DM [20]. A study from Tianjin, China, showed that the prevalence of dyslipidemia in diabetic microvascular complications was 77.1% [12], and the prevalence of dyslipidemia in DR was 79.0% in Sabah [19]. In this study, the prevalence of dyslipidemia was increased in the DKD (88.7%), DR (89.8%) and DPN (90.3%) groups, and it was higher than the former. The lipid levels were increased compared to those in control group. Moreover, the increasing trend in the prevalence of dyslipidemia was pronounced as the number of complications increased, especially in the high TG rate group. All of these results indicated that dyslipidemia was associated with diabetic microvascular complications.

There are close relationships between high TG levels and microvascular complications. Although it has been reported that high TG levels are associated with DKD [7, 12, 18], some studies did not find such an association [21, 22]. Compared with DKD, the associations between DR and levels of TG and HDL-C are weaker [18]. A community-based study by Hua Yang indicated that HDL-C and TG levels were independently related to DKD; but not to DR [7]. Earlier studies found that DR was more associated with TG levels but appeared to have a weaker relationship with HDL-C levels. According to Lyons, the severity of DR is positively correlated with TG levels and negatively correlated with HDL-C levels [23]. The role of hyperglycemia in DPN is well-established. A powerful study reported that a high TG level could increase the risk of DPN, while a high level of TG significantly increases the risk of lower limb amputation [24]. In this study, to further investigate the association between lipid profiles and diabetic microvascular complications, multiple logistic regression analysis was performed. After adjustment for age, sex, duration of T2DM, medical treatment, hypertension, BMI, and HbA1c level, only a high TG level remained a risk factor for DKD, DR, and DPN. The result was similar to those of Sacks, Lyons, Callaghan et al. [18, 23, 24]. In addition, the effect of high TG levels on DKD was more obvious than the effect of high TG levels on DR and DPN. This reason might be related to differences in the pathogenesis of diabetic complications between the different tissues and organs. For the general population, to reduce the risk of cardiovascular morbidity, TG levels should be reduced to 2.26 mmol/L [10]. However, for diabetic patients, TG levels are more strictly controlled, and should be kept below 1.7 mmol/L [1, 25]. In this study, when the TG level was ≥ 1.60 mmol/L, the risk of diabetic microvascular complications significantly increased. The results revealed why diabetic patients should keep their TG level lower. In addition, most T2DM patients suffer from liver steatosis and high blood pressure. The study showed that the TG/HDL ratio was highly correlated with liver steatosis and high blood pressure [26, 27]. Therefore, TG levels should be given more attention to control other complications. Female sex, older age, longer T2DM duration, obesity, and higher HbA1c levels are risk factors for diabetic microvascular complications [28–30], so subgroup analysis was applied to analyze the effect of high TGs in different groups. The results showed that age (years) > 55, T2DM duration (years) > 10, and HbA1c (%) ≥ 7 were more likely to increase the risk of high TGs, which it indicated that patients with older age, longer T2DM duration, and higher HbA1c levels are recommended to keep their lipid levels more strictly.

Although many studies have correlated diabetic microvascular complications with high TG levels, the findings have been highly debated. Moreover, most of the previous studies only analyzed the lipid profiles’ effect on one of the complications. This study collected the DKD, DR, and DPN data, and thus provided a more comprehensive analysis of the relationship between DKD, DR, and DPN and lipid profiles. The results showed high TG level increased the risk of diabetic microvascular complications and reaffirmed the importance of lowering TG concentrations.

### Study strength and limitation

The study indicated that high TG levels had a significant correlation with the risk of DKD, DR, and DPN. However, this study has some limitations. The single-center retrospective study may have cause some data bias in sample selection. Moreover, this cross-sectional design only allowed for the assessment of the associations between lipid profiles and microvascular complications, but not the definitive cause-and-effect relationship. In addition, this study lacked some data including patients’ history of smoking and alcohol consumption, which may have affected the results of the analysis.

## Conclusion

High TG levels increase the risk of diabetic microvascular complications, and the patients with the older age, longer T2DM duration, and higher HbA1c level are recommended to keep their lipid levels more strictly.

## Data Availability

All data generated or analyzed during this study is included in this published article.
